# Resource-Efficient FPGA Architecture for Real-Time RFI Mitigation in Interferometric Radiometers

**DOI:** 10.3390/s24248001

**Published:** 2024-12-14

**Authors:** Adrian Perez-Portero, Jorge Querol, Adriano Camps

**Affiliations:** 1 CommSensLab—UPC, Universitat Politècnica de Catalunya—BarcelonaTech, 08034 Barcelona, Spain; 2 Institute of Space Studies of Catalonia (IEEC)—CTE-UPC, 08860 Castelldefels, Spain; 3 MITIC Solutions S.L., 08017 Barcelona, Spain; 4 Interdisciplinary Centre for Security, Reliability and Trust (SnT), University of Luxembourg, 1855 Luxembourg, Luxembourg; 5 College of Engineering, United Arab Emirates University, Al Ain 15551, United Arab Emirates

**Keywords:** radio frequency interferece, RFI, FPGA, earth observation, interferometric radiometers, polarimetry

## Abstract

Interferometric radiometers operating at L-band, such as ESA’s SMOS mission, enable crucial Earth observations providing high-resolution measurements of soil moisture, ocean salinity, and other geophysical parameters. However, the increasing electromagnetic spectrum utilization has led to significant Radio Frequency Interference (RFI) challenges, particularly critical given the sensors’ fine temperature resolution requirements of less than 1 K. This work presents the hardware implementation of an advanced RFI detection and mitigation algorithm specifically designed for interferometric radiometers, targeting future L-band missions. The implementation processes 1-bit quantized signals at 57.69375 MHz from multiple receivers, employing time-frequency analysis and polarimetric detection techniques while optimizing Field Programmable Gate Array (FPGA) resource utilization. Novel optimization strategies include overclocked processing cores operating at 230.775 MHz, efficient resource sharing through operation serialization, and strategic memory management. The system achieves real-time processing capabilities while maintaining detection probabilities above 63% with false alarm rates below 1% for typical interference scenarios. Performance validation using synthetic datasets demonstrates robust operation across various RFI conditions, making this implementation suitable as part of the RFI detection and mitigation efforts for future interferometric radiometer missions beyond SMOS.

## 1. Introduction

Microwave radiometry has established itself as a cornerstone of Earth Observation (EO) systems, providing precise data for monitoring critical environmental parameters such as soil moisture, ocean salinity, and atmospheric conditions [1]. Current operational satellites employing microwave radiometers, including Soil Moisture and Ocean Salinity (SMOS) [2] and Soil Moisture Active Passive (SMAP) [3], have demonstrated the technology’s capabilities for global environmental monitoring. These instruments operate in protected frequency bands theoretically reserved for passive observations. However, the exponential growth of wireless communications, coupled with unauthorized transmissions and out-of-band emissions, has led to an increase in Radio Frequency Interference (RFI) incidents [4,5,6]. The proliferation of RFI sources compromises data quality and scientific observations, presenting a significant challenge for current and future Earth observation missions. RFI can originate both from external sources and from conducted or radiated interference within the satellite itself [7]. The impact of RFI on radiometric measurements manifests in various forms, ranging from subtle biases that distort scientific data to complete data loss in severely affected regions. Analysis of SMOS mission data has revealed significant RFI contamination patterns, particularly over densely populated regions in Asia and Europe [8]. In some areas, persistent interference has rendered measurements completely unusable, necessitating extensive data filtering and correction procedures [9]. Similar challenges have been documented for the SMAP mission [10] and other radiometric systems, highlighting RFI as a critical concern that must be addressed to ensure the viability of future Earth observation missions [11].

The scientific community has responded to these challenges by developing increasingly sophisticated RFI detection and mitigation strategies [12,13,14]. These approaches have evolved from simple threshold-based techniques to complex multi-domain analysis methods.

**Figure 1:**
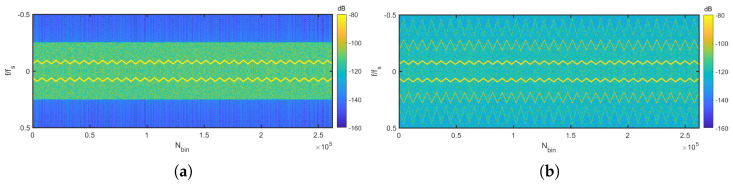
Effects of 1-bit quantization on a 15,000 K chirp RFI signal with thermal noise. (**a**) Original unquantized spectrogram showing the chirp’s natural frequency progression. (**b**) After 1-bit quantization, revealing harmonic generation and aliasing effects, among others.

After the key lessons learnt from SMOS [15], a new architecture for advanced L-band radiometers using 1-bit quantization at a higher sampling rate was proposed [16]. As part of the technology activities, the RFI detection algorithm presented in [17] was developed. The algorithm processes the 1-bit quantized signals and employs innovative techniques such as frequency-domain cross-correlation computation and Polarimetric Kurtosis, offering promising results in theoretical and simulation studies. The challenges of RFI detection with highly quantized data are illustrated in Figure 1, which shows a chirp interference signal before and after 1-bit quantization. The quantization process introduces several critical effects: signal clipping fundamentally alters both temporal and spectral properties, generating harmonics at integer multiples of the RFI’s fundamental frequency, some of which manifest as aliases in the frequency domain. Despite these distortions, the temporal and spectral signatures of the RFI remain detectable, motivating detection algorithms that exploit these preserved characteristics while accounting for quantization effects. Microwave radiometers must detect extremely weak signals, often below −100 dBm [18], making them particularly susceptible to electromagnetic interference. The loss of sensitivity due to the 1-bit sampling, since the hard decision threshold between 0 and 1 means that any interference above the noise floor can directly impact the quantization decision, can potentially corrupt measurements more severely than in systems with higher bit depth that provide amplitude information.

However, the transition from theoretical algorithms to space-qualified implementations presents a formidable set of challenges. Space missions demand highly optimized hardware implementations that must operate reliably for years with minimal intervention. Field Programmable Gate Array (FPGA) implementations must balance complex trade-offs between processing capabilities, resource utilization, and power consumption [19]. Real-time processing requirements necessitate careful optimization of data flow and computational resources, while space operation demands robust error handling and fault tolerance mechanisms [20]. These implementation challenges become particularly hard for interferometric radiometers, where numerous receivers must be processed simultaneously. Some examples of such implementations can be found in [13,21,22] for multi-bit inputs. Conventional approaches often rely on dedicated FPGAs for each receiver or receiver pair, leading to increased system complexity, power consumption, and mission costs. Space-grade FPGAs present additional constraints, typically offering fewer resources than their commercial counterparts while demanding more robust design practices and thorough validation procedures. Implementation considerations extend beyond basic resource allocation to encompass complex system-level challenges. Clock domain management and synchronization become critical when dealing with multiple data streams and processing stages. Fixed-point arithmetic must be carefully designed to maintain precision throughout the processing chain while minimizing resource usage. Memory bandwidth optimization requires strategic buffering and efficient data movement strategies. Pipeline design must balance throughput requirements against resource constraints, often necessitating creative approaches to resource sharing and operation serialization.

The work presented herein addresses these implementation challenges, describing a practical realization of advanced RFI detection and mitigation algorithms specifically optimized for space-based interferometric radiometers explored in [17]. Through innovative approaches to overclocking, resource sharing, and operation serialization, the implementation achieves significant reductions in FPGA resource requirements while maintaining real-time processing capabilities. The design emphasizes reliability and flexibility, allowing parameter adjustment during mission lifetime while maintaining robust operation in the space environment. The following sections detail the development and validation of this implementation. Section 2 presents a comprehensive block design, examining the data flow architecture and processing stages. Section 3 explores implementation optimization strategies, detailing novel approaches to overclocking, serialization techniques, and resource utilization improvements. Section 4 describes the validation methodology, presenting results from synthetic data testing, hardware-in-the-loop validation, and performance measurements. Finally, Section 5 offers conclusions and examines potential pathways for future implementation improvements.

## 2. Algorithm Description

This section describes an RFI detection and mitigation algorithm optimized for interferometric radiometers with 1-bit digitization. The algorithm combines statistical analysis through kurtosis estimation with polarimetric measurements to identify and remove RFI contamination. It processes both time and frequency domains to provide with a wider range of detection opportunities. The detection strategy makes use of the non-Gaussian characteristics of RFI signals and their impact on signal polarization, allowing for effective identification even with highly quantized input data.

**Figure 2:**
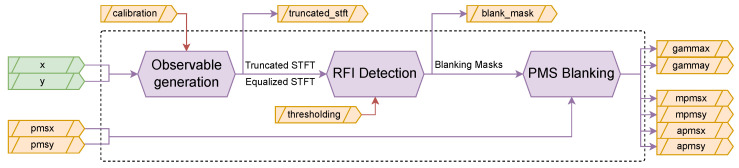
Simplified overview of the RFI Mitigation algorithm implementation showing the three main processing stages and data flow paths. Configurable or external inputs are shown with red arrows.

The Hardware Design Language (HDL) architecture has been structured to minimize resource utilization to allow for multiple receivers to be instantiated in the same FPGA, while maintaining processing capabilities. The RFI Mitigation algorithm implementation is separated in three distinct processing stages (Figure 2), each addressing specific aspects of the detection and mitigation process: first, the Observable Generation stage produces the intermediate products necessary for RFI detection. This stage implements the theoretical framework described in [17], computing the time-frequency representations and polarimetric parameters while managing the strict resource constraints of space-grade FPGAs. The stage handles the critical 1-bit quantized input signals, processing them from the base sampling rate of 57.69375 MHz, through an overclocked Short-Time Fourier Transform (STFT), while preparing the data for subsequent analysis. Second, the RFI Detection stage processes the observables to identify interference in both time and frequency domains. This stage implements the multi-domain analysis approach, generating blanking masks at the sample level. The detection process incorporates both the statistical and polarimetric tests. Third, the PMS Blanking stage applies the generated masks to mitigate RFI in both the 1-bit quantized signals and the Power Measurement System (PMS) data. This final stage ensures the cleaned signals maintain proper synchronization for the subsequent correlation processing.

The implementation adopts a modular architecture where these three processing stages are encapsulated in separate HDL blocks. The design enables controlled data flow management between processing stages, crucial for maintaining real-time processing capabilities. External buffering handles the 3-bit truncated STFT outputs and 1-bit polarization signals, ensuring proper synchronization with the correlator timing requirements. The buffering strategy optimizes memory usage while maintaining the necessary throughput for real-time operation. A key architectural decision involves processing each receiver independently. The design processes a single receiver’s data path, allowing horizontal scaling through multiple instantiations of the RFI Mitigation block. The ability to scale horizontally by replicating processing blocks provides flexibility in adapting the implementation to different mission requirements and hardware constraints.

Resource-intensive operations, particularly the Fast Fourier Transform (FFT) computation and wide-word divisions, have been specifically optimized. The FFT implementation employs serialization techniques and resource sharing strategies to minimize Digital Signal Processing (DSP) block usage while maintaining throughput requirements. Similarly, division operations are optimized through Automatic Gain Control (AGC) units that reduce the width of the operands. Memory management emphasizes the use of Block RAM (BRAM) over distributed memory and registers. This strategy reduces the overall logic element usage while providing the necessary storage capacity for intermediate results and processing buffers. The following sections provide detailed block diagrams and information for each processing stage, examining the specific optimization strategies employed to meet space implementation requirements while maintaining algorithm effectiveness.

### 2.1. Interface Definition

The RFI detection and mitigation system interfaces with multiple data streams and control signals, as illustrated in Figure 2. Each interface serves a specific purpose in the processing chain.

The system’s primary inputs are two 1-bit quantized data streams (*x* and *y*) representing the in-phase and quadrature components from each receiver’s X and Y polarizations. These signals are sampled at 57.69375 MHz and feed directly into the Observable Generation block. In parallel, the system receives PMS measurements (*pmsx*, *pmsy*) at a lower rate of approximately 28 kS/s, which provide total power information for each polarization channel. The final stage applies the generated blanking masks to both the high-speed signal path and the PMS measurements. The outputs include gain-corrected PMS measurements for both polarizations (*gammax*, *gammay*), mitigated and averaged PMS values (*mpmsx*, *mpmsy*), and unmitigated and averaged PMS values (*apmsx*, *apmsy*). These outputs provide both debug and clean measurements for subsequent radiometric processing.

### 2.2. Observable Generation

**Figure 3:**
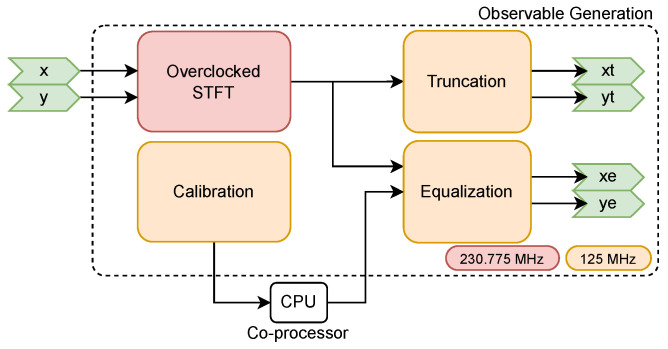
Observable Generation block diagram detailing the processing chain from 1 to bit inputs through windowing and FFT stages to truncated and equalized outputs. The different clock domains are higlighted in red and orange.

The Observable Generation subblock (Figure 3) generates intermediate signals that are then used in the RFI Detection and PMS Blanking subblocks for further processing, as well as the input truncated signals of the advanced correlator. These intermediate signals correspond to the truncated and equalized STFT, obtained from the X- and Y-polarization input signals. This subblock includes a calibration procedure to extract statistical information from the input signals to equalize the generated STFT. The truncated signals can also be configured externally with a truncation factor ($\delta_{p}$). The main purpose of this block is to convert the X- and Y-polarization real and imaginary samples to a suitable format for RFI detection. This intermediate format includes a 3-bit truncated STFT output, and a 16-bit equalized STFT output.

#### 2.2.1. Calibration

The calibration procedure allows the partial computation of the equalization coefficients, with the help of an external processor to perform the costlier steps. By default, *dataEq* is set to 1, thus applying no equalization. In order to obtain the proper values to use in the *dataEq* field, the calibration procedure is necessary. The procedure involves setting *MCalSet* to a number of time slots that want to be integrated. Once *startCal* is set to true, the block will start integrating the Power Spectral Density (PSD) of the data in X and Y. When *MCalSet* time slots have passed (*calDone* is true), the accumulator will be output through the *dataCal* port when *validCal* is true. It is important to note that the real part of this port corresponds to the values for X-pol, and the imaginary part to Y-pol. It is possible to specify an address for this value to be output to, by using the *addrCal* port, together with the *vaddrCal* flag. With this value, the Equalization coefficients can be calculated by dividing them by *MCalSet*, performing the square root and inverting the value. The result of this operation will be set to the *dataEq* port to complete the calibration.

#### 2.2.2. Equalization and Truncation

The 3-bit truncation applied by the Observable Generation block can be configured by means of the $\delta_{p}$ input variable. $\delta_{p}$ is a scaling factor used during truncation, defined as:

(1) $$\delta_{p}=\frac{2^{N_{bits}-1}-1}{A_{clip}}=\frac{2^{N_{bits}-1}-1}{\delta \;\cdot \;\sqrt{2}},$$

where the number of quantization bits is $N_{bits}$ = 3, and the scaling factor is δ = 2 [23]. In principle, δ must be selected so that the clipping effects are negligible. This factor is applied to the signal prior to truncation as:

(2) $$\begin{array}{cc} Xr & =ℜ\left(X\right)\;\cdot \;\delta_{p} \end{array}$$

(3) $$\begin{array}{cc} Xi & =ℑ\left(X\right)\;\cdot \;\delta_{p} \end{array}$$

(4) $$\begin{array}{cc} Yr & =ℜ\left(Y\right)\;\cdot \;\delta_{p} \end{array}$$

(5) $$\begin{array}{cc} Yi & =ℑ\left(Y\right)\;\cdot \;\delta_{p}, \end{array}$$

and it controls the clipping point for the truncation. A deeper study on the effects of the δ parameter can be found in Appendix A of [17].

### 2.3. RFI Detection

**Figure 4:**
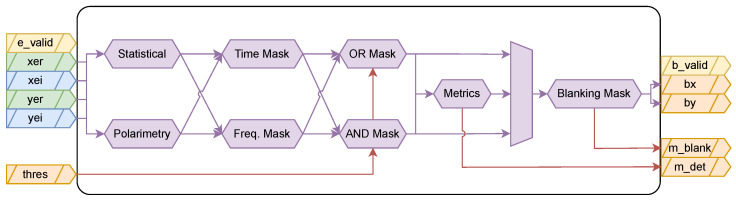
RFI Detection architecture showing parallel computation of statistical and polarimetric parameters in time and frequency domains. The detection logic combines multiple metrics to generate blanking masks for RFI mitigation. Configurable or external inputs, and debug outputs, are shown with red arrows.

The RFI Detection subblock (Figure 4) uses the intermediate outputs generated by the Observable Generation block and creates blanking masks to allow mitigation of the input signals if they are contaminated by RFI. The main inputs of this subblock are the Equalized STFT outputs obtained from the Observable Generation block, and the outputs correspond to metrics on the process and the blanking masks to be used when mitigating the input observables. The thresholds used in the detection of RFI signals can be tuned by changing the Threshold ports, both in time and frequency.

#### Threshold Calculation

The time and frequency thresholds are the detection thresholds for statistical and polarimetry metrics to determine that an RFI signal is present. The value of these thresholds may take two different specific values whether they are used for temporal or spectral moments. For the values K=1024, M=4096, and $PFA=1\;\cdot \;10^{-8}$, the time threshold is 0.3582, and the frequency threshold is 0.1791. These theoretical thresholds are obtained as:

(6) $$\begin{array}{cc} \alpha_{f} & =\sqrt{\frac{4}{M}}\;\cdot \;\sqrt{2}\;\cdot \;{\mathrm{erf}}^{-1}\left(1-\mathrm{PFA}\right) \end{array}$$

(7) $$\begin{array}{cc} \alpha_{t} & =\sqrt{\frac{4}{K}}\;\cdot \;\sqrt{2}\;\cdot \;{\mathrm{erf}}^{-1}\left(1-\mathrm{PFA}\right), \end{array}$$

where $\alpha_{f},\alpha_{t}$ correspond to the frequency and time thresholds, respectively, M,K correspond to the number of samples in the frequency and time domains, respectively, erf is the error function, and PFA is the Probability of False Alarm.

A different threshold, the beta threshold (β) or maximum blanking threshold, is used to adjust the amount of positive detections in the masks that is allowable so as to not excise a significant part of the desired signal. More information on this procedure can be found in Section 2.1.6 of [17]. The RFI mitigation is based on the excision of the contaminated samples out of the set of all transformed samples. The RFI mitigation operates efficiently if few samples contain the largest fraction of the RFI power. However, this may not be the case when the RFI power is well-spread across the time-frequency space. In these cases, it may happen that almost all samples are discarded and, therefore, no signal remains at the output of the RFI mitigation algorithm. The value of β determines which type of mitigation approach is applied to the signal. A typical value for the beta threshold is 1.

### 2.4. PMS Blanking

**Figure 5:**
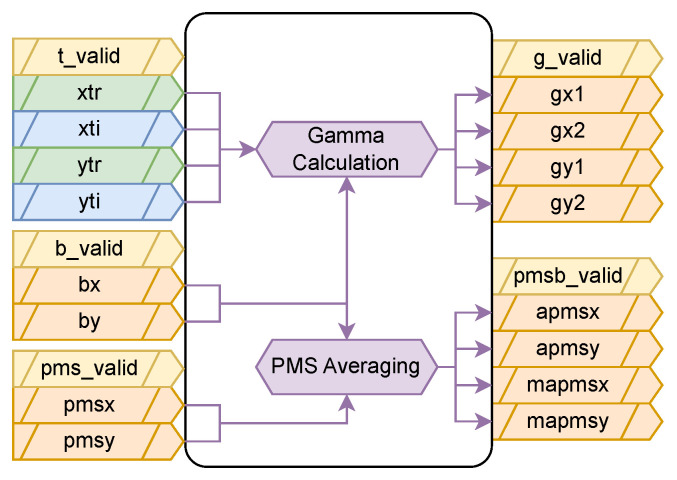
PMS Blanking implementation illustrating the application of blanking masks to both high-speed signals and PMS measurements. Rate conversion and gain correction stages ensure proper synchronization and calibration.

The PMS Blanking subblock (Figure 5) performs the final mitigation of the detected RFI from the input PMS signals, using the masks provided by the RFI Detection subblock, and the Truncated STFT signals provided by the Observable Generation block, to provide the X- and Y-polarization averaged (and mitigated) PMS signals. The output of this block includes the Gamma parameters used to scale the final mitigated signal.

The mitigation of the power measurements follows a pulse blanking approach (mitigation in the time domain), but instead of using just the instantaneous power value to infer the presence of RFI if it is above a given value (typically several times the standard deviation of the power itself, assuming it is RFI-free), the blanking mask is calculated directly from the temporal moments of the polarimetric kurtosis ($b_{x}\left[m\right]$ and $b_{y}\left[m\right]$). The ratios, $\gamma_{x}$ and $\gamma_{y}$, are calculated for each receiver *r*, representing the ratio between the power of the bins after mitigation and before, as:

(8) $$\begin{array}{c} \gamma_{x,r}=\frac{\sum_{m=0}^{M-1}\sum_{k=0}^{K-1}{\left|X_{r}^{mit}\left[m,k\right]\right|}^{2}}{\sum_{m=0}^{M-1}\sum_{k=0}^{K-1}{\left|X_{r}\left[m,k\right]\right|}^{2}}, \end{array}$$

(9) $$\begin{array}{c} \gamma_{y,r}=\frac{\sum_{m=0}^{M-1}\sum_{k=0}^{K-1}{\left|Y_{r}^{mit}\left[m,k\right]\right|}^{2}}{\sum_{m=0}^{M-1}\sum_{k=0}^{K-1}{\left|Y_{r}\left[m,k\right]\right|}^{2}}., \end{array}$$

where $X_{r}^{mit}\left[m,k\right]$ and $Y_{r}^{mit}\left[m,k\right]$ are the different mitigated time and frequency observables for the X and Y polarizations, whereas $X_{r}\left[m,k\right]$ and $Y_{r}\left[m,k\right]$ are unmitigated. These ratios are used to compensate for the bias introduced by the RFI signal into the PMS measurements. Note that, ideally, output PMS signal should be obtained directly from the mitigated STFT if the input signal had multiple quantization bits. After the mitigation of the corrupted PMS, the sum of PMS samples that are not discarded has to be normalized by the gamma factor, in order to estimate properly the power in each channel/polarization, as:

(10) $$\begin{array}{c} P_{x,r}^{mit}=\sum_{b_{x}^{time}\left[m\right]=1}P_{x,r}\left[m\right]\;\cdot \;\gamma_{x,r}, \end{array}$$

(11) $$\begin{array}{c} P_{y,r}^{mit}=\sum_{b_{y}^{time}\left[m\right]=1}P_{y,r}\left[m\right]\;\cdot \;\gamma_{y,r}., \end{array}$$

where $P_{x,r}^{mit}$, $P_{y,r}^{mit}$ are the mitigated powers for the X and Y polarizations and receiver *r*, $P_{x,r}$, $P_{y,r}$ are the unmitigated powers, and the $b_{x}^{time}\left[m\right]=1$ and $b_{x}^{time}\left[m\right]=1$ sums iterate over the blanking masks, where they are equal to 1.

## 3. Implementation Optimization

The implementation of any DSP algorithms in FPGAs requires careful optimization to meet real-time processing requirements while efficiently utilizing available resources. This section discusses the key optimization strategies employed through the implementation of the RFI mitigation algorithm.

### 3.1. Fixed-Point Design

**Figure 6:**
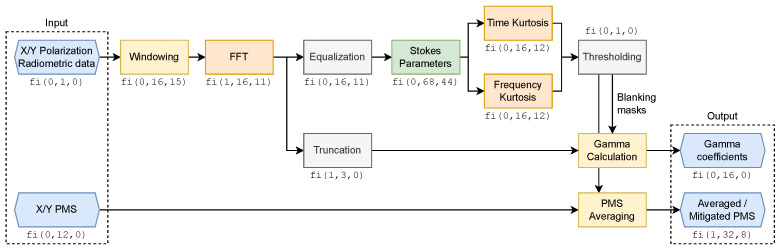
Fixed-point implementation of radiometric data processing chain. The diagram shows bit-width evolution through signal processing stages. Block colors indicate inputs and outputs (blue), lossless processing (gray), and precision loss: green for low, yellow for medium, and orange for significant precision reduction.

The data processing chain (Figure 6) shows characteristic bit-width changes through fixed-point operations. It demonstrates strategic fixed-point scaling choices, with bit-width expansion in multiplication-heavy operations (windowing, Stokes), and controlled reduction in statistical computations (kurtosis) and power measurements (PMS).

As previously introduced, the radiometric signal is quantized at 1 bit. The effects of this choice in terms of radiometric sensitivity have been discussed in the introduction, but conversely, it also offers substantial hardware efficiency advantages. The reduced bit width directly translates to smaller hardware footprints in critical components, including adders, accumulators, and associated routing resources. This architectural choice cascades into practical system-level benefits: decreased memory requirements, optimized FPGA resource utilization, enhanced timing performance through simplified logic paths, and reduced overall power consumption.

The radiometric data processing chain in the Observable Generation block begins with windowing, applying a Hamming window to shape the temporal response for subsequent STFT analysis. The window function reduces spectral leakage and improves frequency resolution, though expanding the word length to fi(0,16,15) due to the multiplication with window coefficients. The FFT stage introduces bit growth proportional to $\log_{2}\left(N\right)$ through its butterfly additions, but after scaling by $\sqrt{N}$, it settles at fi(1,15,11). This follows from the theoretical maximum growth in FFT processing, where the twiddle factor multiplications introduce negative values into the computation. The fraction length is reduced to avoid excessive growth in later stages.

The RFI Detection block implements two key operations: the computation of Stokes parameters and the estimation of spectral kurtosis in both time and frequency domains. The Stokes parameters computation requires careful management of numerical growth through the processing chain. Starting from an initial fixed-point representation of fi(0,16,11) for the input samples, the word length expands significantly due to the successive multiplication operations. The most demanding case occurs in the computation of the $S_{4}$ parameter, where the bit-width grows up to fi(1,68,44) to maintain precision through the complex products. This expansion is necessary to prevent arithmetic overflow and preserve the detection sensitivity across the full dynamic range of the input signals. The most numerically challenging aspect lies in the kurtosis computation, which requires extensive accumulation of fourth-order moments. While this accumulation inherently demands high numerical precision during intermediate calculations, the final kurtosis values are represented using fixed-point format fi(0,16,12). This reduced precision is justified by the detection mechanism itself: kurtosis-based RFI detection relies on threshold comparison rather than precise magnitude estimation. When the kurtosis deviates significantly from its theoretical value for Gaussian signals, indicating the presence of RFI, the exact magnitude of this deviation becomes irrelevant for detection purposes. This implementation consideration significantly reduces hardware resources while maintaining detection effectiveness. Finally, the thresholding stage converts the kurtosis values into binary decisions, resulting in the binary blanking masks.

In the PMS Blanking block, the implementation of gamma calculations (Equations (8) and (9)) is optimized for FPGA resources by separating the numerator and denominator computations, avoiding direct division operations in hardware. Both the numerator and denominator are represented in fixed-point format fi(0,16,0), with the actual division performed in post-processing. This design choice significantly reduces hardware complexity while maintaining the necessary precision for the power ratio estimation. The PMS averaging computation (Equations (10) and (11)) uses a wider fixed-point representation of fi(1,32,8) to accommodate the accumulation of power measurements and ensure sufficient dynamic range for both strong and weak signal conditions. The second path performs the averaging of both the mitigated and unmitigated PMS measurements. The final power estimation is obtained by normalizing the sum of non-discarded PMS samples by their corresponding gamma factors, ensuring accurate power measurements even in the presence of RFI blanking.

### 3.2. Resource Optimization and Throughput Enhancement

The implementation employs serialization of the parallel X/Y polarization inputs, interleaving the data streams before entering the processing chain. By converting parallel data paths into a single serialized stream, the architecture reuses critical blocks including windowing and FFT processors. This serialization strategy reduces FPGA resource utilization by approximately 50% compared to a fully parallel implementation, as identical operations for both polarizations share the same hardware blocks. The resource optimization becomes particularly relevant in FPGA platforms where DSP blocks and memory represent constrained resources.

To maintain processing throughput despite serialization, the system implements a 4x overclocking scheme in the FFT processing block. The input data arrives with a 1/4 duty cycle, providing timing margins that enable clock rate multiplication. By operating the FFT at four times the input clock frequency, the system achieves the same effective throughput as a parallel implementation while utilizing fewer hardware resources. This overclocking strategy ensures that the serialized data path can process both X and Y polarization samples within the required time constraints, matching the performance of a dual-path architecture.

Table 1 provides an estimation of the FPGA resources used by the entire RFI Mitigation block once implemented in the FPGA. The estimation is performed by Vivado with knowledge of the architecture of the FPGA where the block will be implemented in, but is still missing further optimizations that can only be performed once the block is implemented with the rest of the system, instead of isolated.

## 4. Validation and Testing

The validation of RFI detection and mitigation implementations requires a systematic approach to verify both functional correctness and performance under various operating conditions. The testing strategy consists of multiple stages, from synthetic data validation to full system integration testing, ensuring the implementation meets its design specifications while maintaining real-time performance. The simulation was performed either by MATLAB R2022a HDL Coder or MATLAB R2022a Simulink (MathWorks), and the results were compared numerically to the outputs of the original MATLAB algorithm, which uses floating-point double-precision data types. Initial validation employs synthetic datasets designed to test specific aspects of the implementation. Test vectors include:

- Clean radiometric signals with known statistical properties;
- Simulated RFI patterns of varying intensity and temporal characteristics;
- Corner cases designed to stress the fixed-point arithmetic implementation.

The RFI detection performance was evaluated using simulated impulsive interference with varying occurrence probabilities. The RFI was modeled as pulses with amplitude 10× higher than the background thermal noise (modeled as Gaussian white noise), added to both I/Q components of dual-polarization channels. We tested different false alarm probabilities (PFA = 1 × 10^−2^, 1 × 10^−3^, and 1 × 10^−4^) by adjusting the RFI occurrence rate accordingly, allowing assessment of the detection algorithm under different interference scenarios. Results from hardware simulation are compared against high-precision floating-point MATLAB implementations.

**Figure 7:**
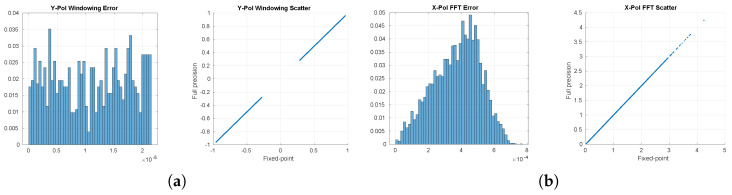
Main sources of precision loss in the Observable Generation block. (**a**) Comparison of the full-precision and fixed-point values after windowing. (**b**) Comparison of the full-precision and fixed-point values after FFT.

The Observable Generation subblock implements signal processing operations that require careful validation to ensure robust RFI detection. The two algorithms in it require special considerations in the implementation phase. The first operation comprises the time-frequency transform operations. The windowing procedure using Hamming window shapes the temporal response and affects spectral leakage characteristics of the subsequent analysis. This windowing stage works in conjunction with the STFT operation, which establishes the time-frequency resolution tradeoff of the system. These operations must maintain numerical stability when processing signals with high dynamic range, even though in this case, the input is 1-bit. To validate the numerical accuracy of the windowing and STFT operations, we compared the output between the full-precision MATLAB implementation and the hardware-oriented Simulink model. Figure 7 shows the error distribution and scatter plot between both implementations, both for the windowing and STFT operations. The close alignment along the diagonal in the scatter plot confirms that the fixed-point implementation maintains adequate numerical precision through the transform stages. For the windowing operation, the implementation achieves a Root Mean Square Error (RMSE) of 1.28 × 10^−5^ with maximum errors of 2.15 × 10^−5^. The subsequent FFT operation shows slightly higher but still acceptable errors, with RMSE values around 3.97 × 10^−4^ and maximum errors below 7.48 × 10^−4^.

The second operation is the Calibration and Equalization stage. The numerical precision in this stage becomes particularly critical when processing strong RFI signals, as they can push the arithmetic operations near their dynamic range limits. Fixed-point implementation considerations directly affect the accuracy of the equalization coefficients. Furthermore, the accumulation of quantization errors through the spectrum integration must be carefully managed to maintain the detection sensitivity required for weak interference signals. The final stage involves bit-width reduction, where the signal is truncated to 3 bits (Equations (2)–(5)). The configuration parameters for this truncation stage (as explained in Section 2.2.2) must be thoroughly validated across the expected range of signal dynamics to ensure reliable operation under all observing conditions. Because these parameters are not fixed, the loss of precision will depend on the final values, and is not included in the validation.

To validate the numerical precision of the kurtosis implementation, the hardware fixed-point results are compared against full-precision computations for both RFI and RFI-free scenarios. Table 2 and Table 3 present the RMSE for the kurtosis of the different Stokes parameters. In the RFI scenario (Table 2), the time-domain kurtosis shows RMSE values of 2.72 × 10^−4^ and 2.81 × 10^−4^ for KX and KY, respectively, while K3 and K4 exhibit slightly lower errors of 2.31 × 10^−4^ and 2.34 × 10^−4^. The frequency-domain implementation shows marginally higher errors, with RMSE values ranging from 7.39 × 10^−4^ to 1.10 × 10^−3^. Similar error patterns are observed in the RFI-free scenario (Table 3), where time-domain errors remain around 2.56 × 10^−4^, confirming that the chosen fixed-point representation maintains consistent precision across different signal conditions.

**Figure 8:**
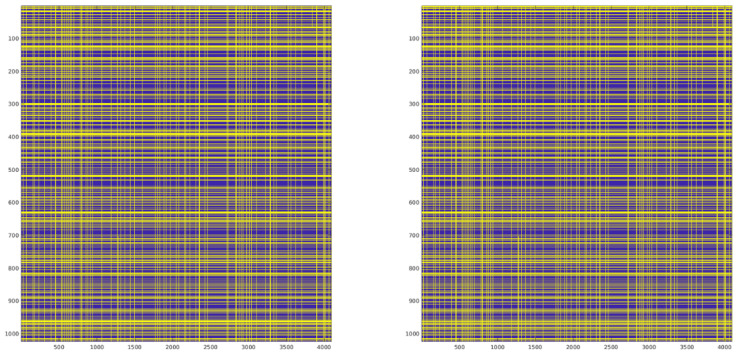
Comparison of the Simulink OR Mask (**left**) with the full-precision MATLAB reference (**right**).

**Figure 9:**
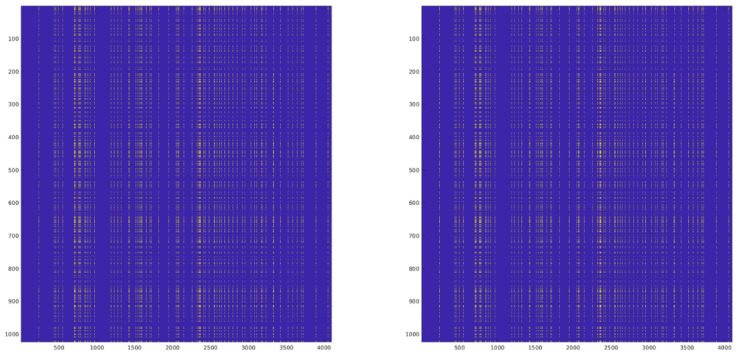
Comparison of the Simulink AND Mask (**left**) with the full-precision MATLAB reference (**right**).

Following the kurtosis computation, detection is performed through thresholding operations (Equation (6)) on each kurtosis estimator ($K_{X}$, $K_{Y}$, $K_{3}$, $K_{4}$). The threshold values are computed following the methodology described in Threshold Calculation Section. Since the thresholding operation is inherently a binary decision, the numerical precision requirements are less stringent than in previous stages. To validate this final stage, we compare the resulting detection masks (AND/OR combinations) between the hardware implementation and the full-precision reference, as seen in Figure 8 and Figure 9. Given the binary nature of these masks, the comparison is performed using Bit Error Rate (BER) measurements rather than Mean Square Error (MSE). This metric directly quantifies the detection performance impact of the complete fixed-point implementation chain, from the initial signal processing through the final detection decision. The BER results (Table 4) provide a practical assessment of the detection reliability, as they represent actual mismatches in RFI identification between both implementations.

The PMS blanking stage maintains measurement accuracy through careful fixed-point design. The process relies primarily on binary operations for sample selection, minimizing computational complexity and precision loss. The gamma coefficients, implemented in 16 bits, derive from temporal and frequency averages of 7-bit values (resulting from squaring and adding the truncated 3-bit values), providing sufficient precision for the blanking masks. This bit width selection ensures negligible precision loss in the coefficient calculation stages. The critical averaging operation of valid PMS samples employs 32-bit arithmetic to preserve precision during the COordinate Rotation DIgital Computer (CORDIC)-based division by the non-constant number of retained samples. This design choice maintains measurement accuracy while normalizing the sum of non-discarded samples.

**Table 4:** Mask error in an RFI scenario (BER).

	X AND	X OR	Y AND	Y OR
RFI Detection contribution	$1.91\times 10^{-6}$	$8.48\times 10^{-4}$	$1.91\times 10^{-6}$	$4.88\times 10^{-4}$
Cumulative error	$3.54\times 10^{-6}$	$1.76\times 10^{-3}$	$2.15\times 10^{-6}$	$8.81\times 10^{-4}$

## 5. Conclusions

The implementation of an RFI detection and mitigation system for interferometric radiometers has been successfully demonstrated, achieving significant optimization of FPGA resources while maintaining detection performance. The Observable Generation stage, through overclocking techniques operating at 230.775 MHz, achieved a reduction in DSP block usage compared to conventional implementations. Resource-sharing strategies in the FFT computation resulted in total DSP utilization of 76 blocks per receiver, enabling the processing of 14 receivers within a single FPGA. Memory optimization through strategic use of BRAM resulted in 968 memory Look-Up Tables (LUTs) per receiver. Resource utilization remained balanced across the FPGA, with maximum BRAM utilization at 3.88%, DSP blocks at 3.96%, and logic elements at 6.80%.

The modular design approach proved particularly valuable during testing and validation, allowing independent optimization of each processing stage. The ability to scale horizontally by replicating processing blocks provides flexibility for future mission requirements, while the standardized interfaces facilitate integration with existing radiometer systems.

The successful implementation demonstrates the feasibility of complex RFI mitigation algorithms in FPGA hardware, providing a pathway for future interferometric radiometer missions. The achieved balance between processing capability and resource utilization establishes a baseline for similar implementations, while the modular architecture ensures adaptability to evolving mission requirements. This implementation provides a foundation for future development of RFI mitigation systems, particularly in scenarios where resource optimization and reliability are of great importance.

## Figures and Tables

**Table 1:** Resource utilization compared with the available resources in the Xilinx KU040 architecture.

	LUT Logic	LUT Memory	Latch	BRAM	DSP
ObsGen STFT	3838	785	7388	7	16
ObsGen EqTrunc	625	0	743	6.5	12
RFI Det	9260	99	5782	30	48
PMS Blank	2755	84	2631	3	0
Total	16,478	968	16,544	46.5	76
KU040	242,400		484,800	1200	1920
Percent	6.80%		3.41%	3.88%	3.96%

**Table 2:** Kurtosis error in an RFI scenario (RMSE).

	KX	KY	K3	K4
Time-domain	$2.72\times 10^{-4}$	$2.81\times 10^{-4}$	$2.31\times 10^{-4}$	$2.34\times 10^{-4}$
Frequency-domain	$7.29\times 10^{-4}$	$7.39\times 10^{-4}$	$1.10\times 10^{-3}$	$1.20\times 10^{-3}$

**Table 3:** Kurtosis error in a no-RFI scenario (RMSE).

	KX	KY	K3	K4
Time-domain	$2.56\times 10^{-4}$	$2.59\times 10^{-4}$	$2.36\times 10^{-4}$	$2.33\times 10^{-4}$
Frequency-domain	$6.24\times 10^{-4}$	$6.75\times 10^{-4}$	$1.09\times 10^{-3}$	$1.24\times 10^{-3}$

## Data Availability

The datasets presented in this article are not readily available.

## Glossary

AGC: Automatic Gain Control; BER: Bit Error Rate; BRAM: Block RAM; CORDIC: COordinate Rotation DIgital Computer; DSP: Digital Signal Processing; EO: Earth Observation; FFT: Fast Fourier Transform; FPGA: Field Programmable Gate Array; HDL: Hardware Design Language; LUT: Look-Up Table; MSE: Mean Square Error; PMS: Power Measurement System; PSD: Power Spectral Density; RFI: Radio Frequency Interference; RMSE: Root Mean Square Error; SMAP: Soil Moisture Active Passive; SMOS: Soil Moisture and Ocean Salinity; STFT: Short-Time Fourier Transform.
